# Testing the influence of habitat experienced during the natal phase on habitat selection later in life in Scandinavian wolves

**DOI:** 10.1038/s41598-019-42835-1

**Published:** 2019-04-25

**Authors:** Cyril Milleret, Andrés Ordiz, Ana Sanz-Pérez, Antonio Uzal, David Carricondo-Sanchez, Ane Eriksen, Håkan Sand, Petter Wabakken, Camilla Wikenros, Mikael Åkesson, Barbara Zimmermann

**Affiliations:** 1 Faculty of Environmental Sciences and Natural Resource Management, Norwegian University of Life Sciences, NO-1432 Ås, Norway; 2 Faculty of Applied Ecology, Agricultural Sciences and Biotechnology, Inland Norway University of Applied Sciences, Evenstad, NO-2480 Koppang, Norway; 3 School of Animal, Rural and Environmental Sciences, Nottingham Trent University, Brackenhurst, Southwell, Nottinghamshire NG25 0FQ UK; 4 Biodiversity and Animal Conservation Lab, Forest Sciences Center of Catalonia (CTFC), Solsona, Spain; 5 Grimsӧ Wildlife Research Station, Department of Ecology, Swedish University of Agricultural Sciences, SE-730 91, Riddarhyttan, Sweden

**Keywords:** Behavioural ecology, Conservation biology

## Abstract

Natal habitat preference induction (NHPI) occurs when characteristics of the natal habitat influence the future habitat selection of an animal. However, the influence of NHPI after the dispersal phase has received remarkably little attention. We tested whether exposure to humans in the natal habitat helps understand why some adult wolves *Canis lupus* may approach human settlements more than other conspecifics, a question of both ecological and management interest. We quantified habitat selection patterns within home ranges using resource selection functions and GPS data from 21 wolf pairs in Scandinavia. We identified the natal territory of each wolf with genetic parental assignment, and we used human-related characteristics within the natal territory to estimate the degree of anthropogenic influence in the early life of each wolf. When the female of the adult wolf pair was born in an area with a high degree of anthropogenic influence, the wolf pair tended to select areas further away from humans, compared to wolf pairs from natal territories with a low degree of anthropogenic influence. Yet the pattern was statistically weak, we suggest that our methodological approach can be useful in other systems to better understand NHPI and to inform management  about human-wildlife interactions.

## Introduction

Animal decision making, such as the selection of home ranges, can be influenced by natal conditions and early phases of learning^1,2^. These conditions, including e.g. social relationships and early life environment have also been shown to influence individual fitness at a later stage of life^3–5^. The habitat use of a given generation may also be influenced by the choices made by the previous generation^6,7^. The effect of natal habitat on habitat selection preferences has generally been termed “natal habitat preference induction” (hereafter, NHPI)^2^. This hypothesis predicts that individuals are more likely to select habitats with characteristics similar to those of their natal territory, possibly as an effective way to find a suitable habitat. NHPI has been demonstrated in several taxa, including mammals such as the brush mice *Peromyscus boylii*^8^ and the Mount Graham red squirrel *Tamiasciurus hudsonicus grahamensis*^9^.

Habitat selection is a hierarchical process occurring at several spatiotemporal scales, where the individual is constantly facing the challenge to select suitable habitat to maximize its fitness^10^. This hierarchical process occurs at four spatial scales, namely first order selection at the distribution range scale, second order selection at the individual home range scale, third order selection at the patch scale within a home range, and the fine-scaled fourth order selection for a specific site or item within a patch^11^. It has been suggested that the most limiting factors should be selected/avoided at the broadest spatial scale^12^. Despite receiving mixed support^10^, this idea implies that the habitat selection at a given spatial scale is the result of constraints of habitat selection at other spatial scales^10^.

Even if characteristics of the natal habitat could influence habitat preferences at the second order, an individual may also show preferences for the stimuli experienced in its natal territory at the third or fourth orders, e.g., when selecting a habitat patch within its home range. However, the influence of early-life conditions on habitat selection has been related mostly to the dispersal and home range establishment^2^. Indeed, Miller *et al*.^13^ highlighted that the potential effects of natal experience on habitat selection after dispersal and territory establishment have received surprisingly little attention, in spite of being the longest period in the life of an individual and the most important for its fitness. Miller *et al*.^13^ is one of the very few studies addressing this potential effect, but see^14^. They found that the natal social environment altered foraging patch decisions of adult females of cactus bug, *Chelinidea vittiger aequoris*, i.e., females reared alone were more likely to feed further from conspecifics than females raised in social environments^13^.

Regarding vertebrates, the preference for natal habitat types has been used as a factor possibly explaining behavioral adaptations by foxes *Vulpes vulpes* to exploit urban habitats, i.e., foxes born and raised in urban areas were more likely to use those areas later in life than other foxes^15,16^. However, the relation between fox behavior and NHPI was not tested. In birds, snail kites *Rostrhamus sociabilis* were more likely to nest in wetlands with similar characteristics to their natal wetland, regardless of the dispersal distance^17^. Interestingly, dispersing kites that bred in natal-like habitats had lower nest success and productivity than kites that chose different habitats^17^. This indicates that natal environments can have long-term effects on the individual, even in highly mobile and wide-ranging animals^17–19^, and it also illustrates that NHPI does not guarantee optimal habitat selection.

For large carnivores, less than a handful of studies have investigated NHPI^19–21^. These studies tested the NHPI hypothesis during home-range establishment, and showed contrasting results. Because large carnivores are highly cognitive species, it might be expected that different types of early experiences may play a role later in life, beyond the potential effect on dispersal and home-range establishment. For example, humans can be an important source of disturbance and pose a mortality risk for large carnivores^22,23^, which generally results in the avoidance of humans, anthropogenic infrastructures and areas of high human activity e.g.^24^. Therefore, experiences with humans during the natal phase may be a stimulus that reflects on carnivore behavior and habitat selection throughout the whole life of an individual.

The Scandinavian wolf *Canis lupus* inhabits human-modified landscapes in which human induced mortality is high^22,23^. Probably as a consequence, wolves consistently avoid human features at different spatial scales^25,26^. Here we tested whether wolves that were exposed to environments characterized by different degrees of anthropogenic influence during their natal phase expressed different habitat selection patterns once they successfully dispersed, i.e., after they settled a territory. Beyond advancing our knowledge on large carnivore ecology, this question also raises management interest. Indeed, this study emerges after the call made by the Norwegian Environment Agency^27^, which showed interest explaining the wolves’ use of areas close to human settlement and infrastructure, as the presence of such behavior may ultimately result in conflict with humans. In line with this call, we advance into the presently scarce knowledge on NHPI on large carnivores.

**Figure 1:**
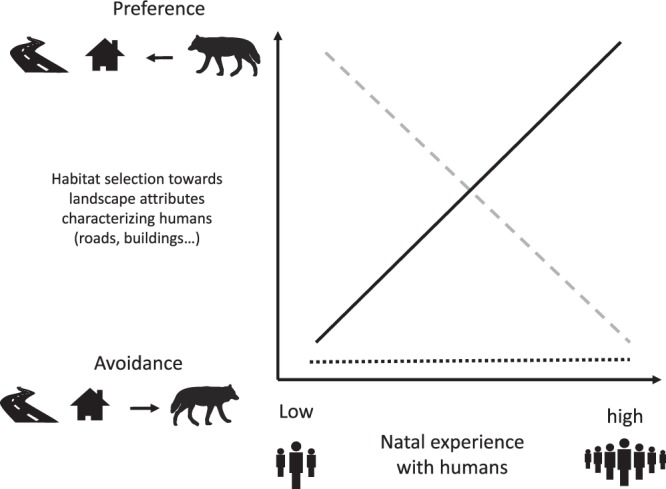
Description of different responses in habitat selection patterns of adult wolves towards human-related landscape features expected under different degrees of natal experience with humans. The negative dashed grey line describes cases where individuals that had negative experience with high level of human activities during their natal phase would avoid humans later in life. The positive black solid line describes cases where individuals that had extensive experience with humans during their natal phase would become less shy towards humans and select areas closer to humans later in life (consistent with NHPI). The horizontal dashed black line describes cases where avoidance of human features is relatively high and independent of experience with humans. All figures were made by the authors.

We proposed three alternative hypotheses regarding the mechanisms behind the individual variation in wolves’ habitat selection relative to human structures of the landscape within their adult home range (Fig. 1):

First, we assumed that wolves born in areas with high human activity are more likely to have experienced close, maybe negative encounters with humans during early life stages, and thus, avoid human activity and human related features of the landscape during adulthood. In contrast, wolves exposed to a low degree of human activity during their natal phase may show selection of human-related features as they are less likely to have had experiences with humans, but may have benefited from them; e.g., using roads for traveling and/or foraging on anthropogenic food sources (Fig. 1, dashed grey line).

The second hypothesis suggests that wolves show NHPI and thus select habitat features within their adult home range that are similar to their natal habitat. As a result, individuals exposed to a high degree of human activity during their natal phase may select for human related features at adult age whereas wolves exposed to a low degree of human activity during their natal phase may avoid human related features at adult age, as these features are unfamiliar (Fig. 1, black solid line).

The last hypothesis suggests that adult wolves’ avoidance of human features is relatively inflexible to natal exposure because human avoidance is an innate behavior that has evolved as a response to historically high rates of human-caused mortality and reduced fitness for individuals living close to humans^28,29^ (Fig. 1, flat black dashed line). The degree of socialization required for captive wolves to lose their shyness towards humans indicates that this fear may involve a genetic component^30,31^.

This study lies at the interface between ecology and management and may prove to be useful for wildlife managers in Scandinavia and elsewhere. Several populations of large carnivores are presently recolonizing human-dominated landscapes (e.g., Bruskotter and Shelby^32^ in North America, Chapron *et al*.^33^ in Europe). Therefore, understanding habitat selection and habitat use of carnivores in areas close to people is increasingly needed by management agencies trying to facilitate long term human-carnivore coexistence.

## Material and Methods

### Study area

**Figure 2:**
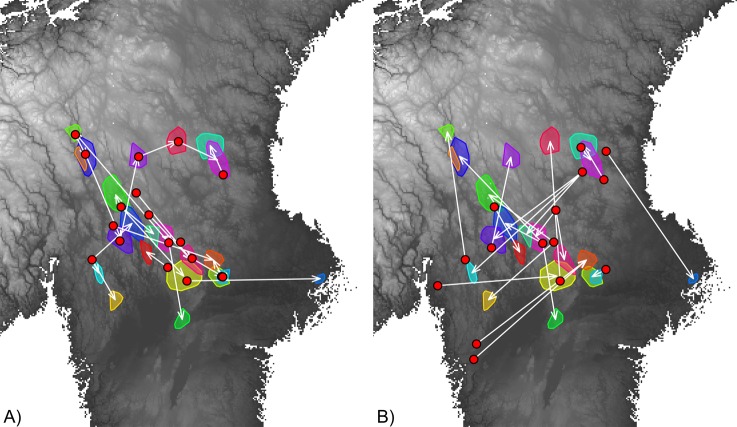
Dispersal maps of the studied females (**A**) and male (**B**) wolves in central Scandinavia from 2001–2015. The white arrows link the location of the centroid of a natal territory (represented by a red dot) to the centroid of the home range of the individual once settled and paired (represented by a colored 100% minimum convex polygon). White to grey background maps represent high to low elevation, respectively.

The study area was located in south-central Scandinavia, within the wolf breeding range (Fig. 2). Habitat types are dominated by boreal coniferous forest, intersected with bogs and lakes. The main tree species are Norway spruce *Picea abies*, Scots pine *Pinus sylvestris*, and birches *Betula pendula* and *B*. *pubescens*^34^. Although average human density within the wolf range is low (with <1 inhabitant/km^2^ in large areas^35^), the density of gravel roads is high due to intensive forest management practices (4.6 times higher than paved road density, which was 0.19 ± 0.02 km/km^2^). Moose *Alces alces* and to a less extent, roe deer *Capreolus capreolus* are the staple prey species for most Scandinavian wolf packs^36,37^.

### Studied animals

#### GPS locations

We obtained detailed information about the habitat selection pattern of adult wolves within their home ranges using GPS^38^ data. From 2001 to 2015, 21 wolf pairs were captured and equipped with GPS–GSM neck collars (VECTRONIC Aerospace GmbH, Berlin, Germany) following ethically-approved veterinary procedures (described in Arnemo *et al*.^39^). The studied wolves were successful dispersers, i.e., territory-holding pair members. We specifically used GPS locations that were recorded at high frequency (generally 30 to 60-min intervals) often for the purpose of intensive predation studies^36,37,40^. Because wolf pairs spend most of their time together except during the reproduction period^41^, we only retained GPS data from the pair member with the largest number of GPS locations for the analysis while still referring to wolf pair locations.

#### Natal territory and pedigree information

Norwegian and Swedish wildlife management authorities conduct extensive wolf monitoring based on snow tracking every winter, where observations of territorial scent markings and estrous blood are recorded and collected to locate and distinguish wolf territories^23,42,43^. DNA analyses allowed the reconstruction of a near complete pedigree of the population, based on invasive samples, including tissues from retrieved dead wolves and blood from captured wolves, and non-invasive samples, including primarily scats and hair^43^. The parental identity of GPS collared individuals was determined from genetic parentage assignment, and the location of the natal territory was then inferred based on the positions of DNA samples and associated snow tracks from the identified parents in their established territory. When the parents were observed during several winters, we used all available locations of the parental pair to calculate the center of the natal territory, since the year of birth of GPS collared offspring was not always possible to determine. We then used a buffer of the average wolf home range size (1000 km^2^; Mattisson *et al*.^44^) around each territory center to approximate the area occupied by the natal territory^23,26^.

### Role of natal habitat in habitat preferences of adult wolves

We followed three steps to estimate the role of natal habitat on habitat preferences of adult wolf pairs. First, we quantified habitat selection of individual wolves within their home range (third order habitat selection) using GPS data and resource selection functions (hereafter RSF; Manly *et al*.^45^). Secondly, we identified the natal territories using the pedigree information and determined the main habitat types in these, thereby representing the habitats experienced by individuals before dispersal. Third, we used linear mixed models to quantify the influence of natal conditions on the habitat selection of wolf pairs once they have successfully dispersed and established elsewhere.

#### Habitat selection

In order to quantify the habitat selection of wolf pairs, we first estimated the home ranges of each pair. Because the periods with intensive GPS locations were limited in time (Supplementary Information 1, Table S1.1), the true home range size was likely underestimated^44^. We therefore defined availability by creating a 100% minimum convex polygon (MCP). We then sampled available locations randomly (20 times the number of used locations) within the MCP for each studied wolf pair. To test the influence of the method chosen to define availability, we also performed the same analysis using 99% kernel home range using the “href” method. Because seasonality was shown to be important for wolf habitat selection^46^, we separated between winter (December 1^st^-April 30^th^) and spring/summer (May 1^st^-July 31^st^) habitat selection.

We extracted the habitat characteristics of used and available locations using different habitat variables that are known to affect wolf habitat selection^25,26,46^ (Supplementary Information 2, Table S2.1). Because our goal was to quantify variation in wolf behavior towards humans, we created two spatial variables representing anthropogenic attributes of the landscape. First, we created a variable that represented the distance from the closest main and gravel roads and buildings (“Distance to all human features”). Second, we only considered distance from the closest main roads and buildings (“Distance to main human features”). We excluded secondary roads because wolves have been shown to use them for traveling, and may therefore show ambivalent selection patterns towards this human feature^25^.

We used a RSF to quantify habitat selection patterns of wolf pairs. We fitted a logistic regression for each wolf pair with the response variable being the used locations (1: GPS locations) and available locations (0: random locations within individual home range). As we aimed at capturing individual variation in habitat selection patterns, we performed a separate RSF for each wolf pair-season^47^.

We built a full model with variables that have previously been shown to affect wolf habitat selection (Supplementary Information 2, Table S2.1), and added alternatively the “Distance to all human features” or “Distance to main human features” variables. Performing a RSF for each wolf pair gave us a proxy for selection/avoidance of human landscape features for each wolf pair. We checked for collinearity among explanatory variables and removed variables with Pearson correlation coefficients >0.6 (Supplementary Information 2, Table S2.1).

Habitat selection may be context dependent. For example, wolves can select secondary roads for traveling, but not for resting^25^. Wolves spend a large part of their time resting and handling prey, but we cannot distinguish these different behaviors from GPS locations only^48,49^. However, we can distinguish when wolves are traveling, which usually represent about 20% of their time spent^48^. We repeated the RSFs as described above, but only retaining the GPS locations when individuals were traveling, to analyze if the potential influence of natal conditions reflected on adult habitat selection in relation with a particular behavior. We defined traveling GPS locations when the speed from the previous position was >200 m/hour. This represents a distance that is equivalent to the predation study protocol used by the Scandinavian Wolf Research Project, to define clusters of GPS locations^48^.

#### Natal conditions

We do not know the mechanisms by which wolves may be influenced by their natal experience, thus we chose to characterize the overall degree of anthropogenic influence to which wolves may have been exposed during their natal phase. Additionally, we did not have access to fine-scale data about the space used by an individual during its natal phase. We therefore extracted the habitat characteristics of the natal territory from a buffer area of 1000 km^2^ (average wolf home range size, Mattisson *et al*.^44^) around the centroid of the natal territory. We only considered human-related characteristics of the landscape, as we were interested in quantifying variation in human exposure within the natal territory of each studied wolf (Supplementary Information 2, Table S2.2). Based on these characteristics we performed a Principal Component Analysis (PCA) on the matrix containing the environmental variables characterizing the natal territories. We then used the score obtained on the main axis of the PCA as an index of exposure to humans during the natal phase.

#### Relationship between habitat selection and natal conditions

We used linear mixed models^50^ in order to investigate the relationship between the adult habitat selection patterns relative to humans (obtained with the RSF) and the habitat characteristic of the natal territory (obtained with the PCA).

We repeated two sets of analyses, with the response variables being the beta coefficients obtained with the RSF for the variables “Distance to all human features” and “Distance to main human features”, respectively. We repeated this analysis for all GPS locations and for GPS locations of wolves while traveling. We used the score of the main PCA axis as the main predictor. We used the ID of the wolf pair as random intercept because some pairs were monitored over several seasons/years. Because a female and a male forming the wolf pair may have experienced different degrees of exposure to humans within their natal territory, we tested whether the natal experiences from the female (“Natal_F”), the male (“Natal_M”), or both (Natal_M + Natal_F and Natal_M* Natal_F) influenced the habitat selection patterns of the wolf pair. Because moose is the main prey of wolves in Scandinavia, we controlled for moose density as a potential confounding factor by including moose density as a covariate in all models. Moose density was extracted from harvest statistics (collected at the moose management unit in Sweden and municipality level in Norway) as the average number of moose shot per km^2^ within the wolf pair home range with a one-year time lag^23,26,51^. We also used the season, late winter (1 March–30 April) – spring (1^st^ may – 30^th^ of June) as a covariate. We then selected the most parsimonious model of the best ranked models according to their Akaike’s Information Criterion (AIC) (Zuur *et al*.^52^, Burnham and Anderson^53^). We considered models within two AIC units to be equally good^53^. All analyses were conducted using R 3.3.3^54^

## Results

### Habitat selection

Wolf pairs generally avoided human features; 40 of 45 wolf pairs had positive coefficients for distance to main human features (Supplementary Information 3, Table S3.1) and selected for forested areas, i.e., 40 of 45 wolf pairs had positive coefficients for forest (Supplementary Information 3, Table S3.1), in line with previous findings for Scandinavian wolves and other large carnivore populations inhabiting human-dominated landscapes.

### Natal conditions

**Figure 3:**
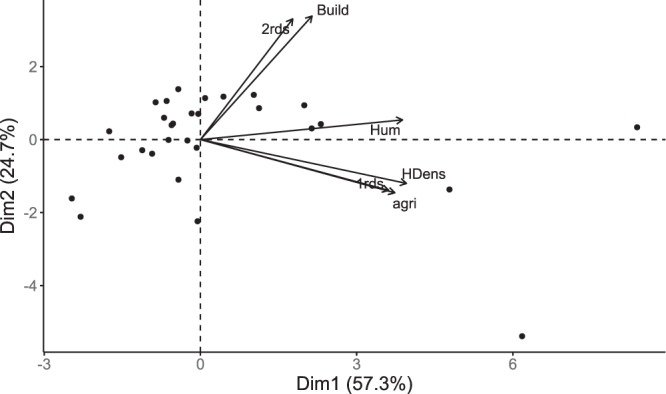
Scores of the natal territories of the studied wolves (in central Scandinavia from 2001–2015) on the first and second axes (“Dim1 and “Dim2”, respectively) of the Principal component analysis. “2rds”: density of secondary roads; “Build”: building density; “Hum”: Proportion of anthropogenic areas; “1rds”: main road density; “HDens”: Human density; “agri”: Proportion of agricultural landscape types.

The first axis of the PCA explained 57.3% of the variation and was retained for the analyses. This axis distinguished between natal territories characterized by high (positive values) versus low (negative values) degree of anthropogenic influence (Fig. 3). The second axis opposed natal territories with high versus low density of secondary roads and buildings, but could not be used to test our hypothesis as it did not characterize areas with high versus low human influence.

**Figure 4:**
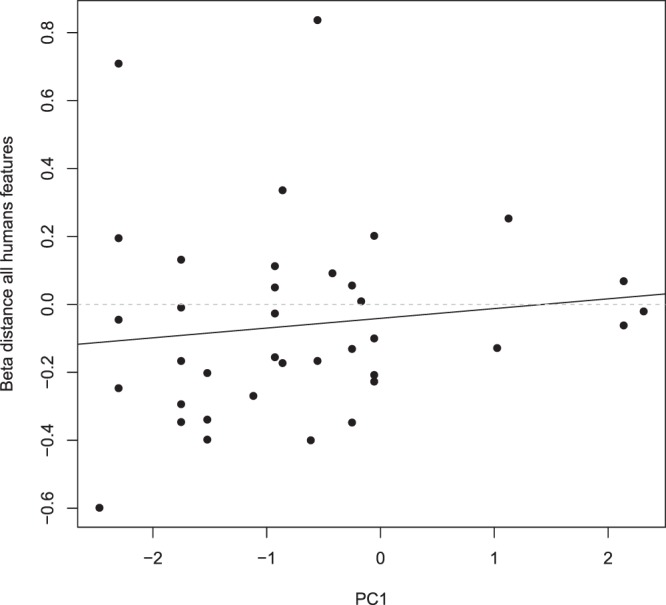
Relationships between the wolf pair behavior towards the “Distance to all human features” variable for all GPS locations of the studied wolves in their adult home range in central Scandinavia from 2001–2015 and their exposure to anthropogenic influence in the natal territory (PC1, positive and negative values denote high and low degree of anthropogenic influence, respectively). A positive beta suggests avoidance of areas close to human features (i.e. human feature is a distance covariate, which denotes selection for areas far from human features).

**Figure 5:**
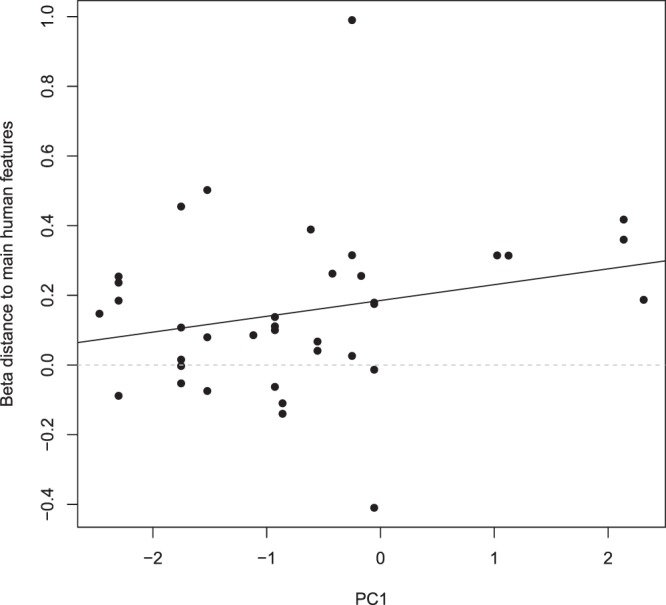
Relationships between the wolf pair behavior towards the “Distance to main human features” variable (excluding secondary roads) for GPS locations of the studied wolves while traveling in their adult home range in central Scandinavia from 2001–2015 and their exposure to anthropogenic influence in the natal territory (PC1, positive and negative values denote high and low degree of anthropogenic influence, respectively). A positive beta suggests avoidance of areas close to human features (i.e. human feature is a distance covariate, which denotes selection for areas far from human features).

Regardless of the method used to define availability (MCP/kernel), and whether we included all of the wolf GPS locations or only the positions while traveling, the null models had the lowest AIC values, except when modelling the selection towards distance to all human features including only the traveling positions (Table 1). Nevertheless, models including the human characteristics of the natal territory of the wolves were always among the best models (delta AIC < 2), and this seemed to be clearer for females than for males (Tables 1 and 2). The beta coefficient for *Natal_F* was always positive, suggesting that wolf pairs composed of a female born in an area characterized by a high degree of anthropogenic influence tended to avoid areas close to humans within their home ranges (Figs 4 and 5, Supplementary Information 4). One of the supported models suggested that this effect was slightly more pronounced in winter than in summer (Table 1, Figs 4 and 5), even though the seasonal effect was very weak and not retained in any other candidate model with delta AIC < 2 (Tables 1 and 2). For males, the regression coefficients and their sign were less conclusive (Supplementary Information 4).

**Table 1:** AIC model selection table for the test of the relationship between human characteristics of the natal territory (obtained from the principal component analysis (PCA)) and the habitat selection of adult wolves in central Scandinavia (from 2001–2015) towards humans (using all GPS locations) after wolves settled as a pair.

Model	AIC	LL	df	deltaAIC	Model	AIC	LL	df	deltaAIC
MCP					KERNEL				
***Distance to all human features***									
Null	13.01	−2.5	29	0	Null	22.26	−7.13	33	0
Natal_F * Season	13.37	0.32	26	0.36	Natal_F	23.83	−6.91	32	1.57
Natal_M	14.94	−2.47	28	1.93	Natal_M	24.2	−7.1	32	1.94
Natal_F	15.01	−2.5	28	2	Natal_M + Natal_F	25.83	−6.91	31	3.57
Natal_M * Season	16.05	−1.03	26	3.05	Natal_F * Season	25.84	−5.92	30	3.59
Natal_M + Natal_F	16.93	−2.47	27	3.92	Natal_M * Season	26.84	−6.42	30	4.58
Natal_M*Natal_F	18.5	−2.25	26	5.49	Natal_M*Natal_F	27.2	−6.6	30	4.94
***Distance to main human features***									
Null	2.55	2.72	29	0	Null	22.2	−7.1	33	0
Natal_F	3.86	3.07	28	1.31	Natal_F	23.64	−6.82	32	1.44
Natal_M	4.43	2.78	28	1.88	Natal_M	24.19	−7.1	32	2
Natal_M + Natal_F	5.28	3.36	27	2.73	Natal_M + Natal_F	25.58	−6.79	31	3.38
Natal_M*Natal_F	7.28	3.36	26	4.73	Natal_F * Season	27.51	−6.76	30	5.31
Natal_F * Season	7.66	3.17	26	5.11	Natal_M*Natal_F	27.55	−6.78	30	5.35
Natal_M * Season	8.26	2.87	26	5.71	Natal_M * Season	27.64	−6.82	30	5.44

Results are presented for selection towards Distance to all human features (includes distance to the closest main and secondary roads, and buildings; top rows) and Distance to main human features (includes distance to the closest main roads and buildings; bottom rows), and when defining availability using minimum convex polygon (MCP, left column) and kernel (right column) for the natal home range. Natal_F and Natal_M correspond to the score of the natal territory obtained on the first axis of the PCA for the female and male, respectively. Season corresponds to summer/winter. All models included moose density as an explanatory variable.

**Table 2:** AIC model selection table for the test of the relationship between human characteristics of the natal territory (obtained from the principal component analysis (PCA)) and the habitat selection of adult wolves in central Scandinavia (from 2001–2015) towards humans (using only GPS locations while traveling) after wolves settled as a pair.

Model	AIC	LL	df	deltaAIC	Model	AIC	LL	df	deltaAIC
MCP					KERNEL				
***Distance all human features***									
Null	14.41	−3.21	36	0	Null	15.29	−3.64	36	0
Natal_F	15.84	−2.92	35	1.42	Natal_F	16.77	−3.38	35	1.48
Natal_M	16.41	−3.2	35	2	Natal_M	17.26	−3.63	35	1.97
Natal_M + Natal_F	17.68	−2.84	34	3.27	Natal_F * Season	18.07	−2.04	33	2.78
Natal_F * Season	17.95	−1.98	33	3.54	Natal_M + Natal_F	18.53	−3.27	34	3.24
Natal_M * Season	18.49	−2.24	33	4.07	Natal_M * Season	18.82	−2.41	33	3.53
Natal_M*Natal_F	19.25	−2.62	33	4.83	Natal_M*Natal_F	20.24	−3.12	33	4.95
***Distance main human features***									
Natal_F	0.89	4.55	35	0	Null	−1.9	4.95	36	0
Null	1.13	3.43	36	0.24	Natal_F	−0.75	5.37	35	1.15
Natal_M + Natal_F	2.68	4.66	34	1.78	Natal_M	0.06	4.97	35	1.96
Natal_M	3.07	3.46	35	2.18	Natal_M + Natal_F	0.81	5.6	34	2.71
Natal_F * Season	3.94	5.03	33	3.04	Natal_M*Natal_F	2.73	5.63	33	4.63
Natal_M*Natal_F	4.66	4.67	33	3.77	Natal_F * Season	2.99	5.5	33	4.89
Natal_M * Season	6.53	3.74	33	5.64	Natal_M * Season	3.75	5.12	33	5.66

Results are presented for selection towards Distance to all human features (includes distance to the closest main and secondary roads, and buildings; top rows) and Distance to main human features (includes distance to the closest main roads and buildings; bottom rows), and when defining availability using minimum convex polygon (MCP, left column) and kernel (right column) for the natal home range. Natal_F and Natal_M correspond to the score of the natal territory obtained on the first axis of the PCA for the female and male, respectively. Season corresponds to summer/winter. All models included moose density as an explanatory variable.

## Discussion

Our results provide some evidence, yet statistically weak, for a relationship between the characteristics of the natal territory of a large carnivore and its habitat selection patterns within its adult home range. Scandinavian wolf pairs tended to use areas close to humans within their home ranges less if their natal territories were characterized by higher human encroachment, as compared to wolves born in areas with a lower degree of anthropogenic influence. The influence of the natal characteristics of the territory seemed more pronounced for females than for males. The pattern was weak, because models containing the natal characteristics of the territory were just as supported as the null model, which had generally the lowest AIC score. Nevertheless, the results remained consistent across models using all or only GPS locations while traveling, and with habitat availability defined with minimum convex polygons or kernel techniques.

The study on a sub-social invertebrate by Miller *et al*.^13^ showed that early experiences could affect adult habitat selection decisions, indicating that such effects may occur in other species. For mammals, it has been argued that a preference for habitat types similar to the natal ones might explain behavioral adaptation of foxes staying in and exploiting the urban habitats where they were born^15,16^. However, whether or not NHPI is a mechanism involved in fox distribution and habitat selection was not tested, and we are not aware of any study confirming a relationship between natal habitat characteristics and third or fourth order habitat selection after dispersal and home range establishment. Therefore, our results, suggesting a relationship between natal conditions and habitat selection within adult wolf home ranges could be considered as novel.

We still know very little about the mechanisms behind behavioral traits, like human avoidance. It might be that human avoidance is a genetically inherited trait that has evolved as a response to selection against traits causing wolves to gravitate towards human activity, as suggested for several bird species^28,29^, and natal conditions may tone that avoidance behavior further. Indeed, avoidance of human-related features may be influenced by learning prior to independence^6^. Still, heritability of fear of humans has been suggested to exist in several bird species^28,29^. The degree of socializing required for captive wolves to lose their shyness towards humans strongly indicates that this fear of humans has a genetic component in wolves as well^30,31^. Because most large carnivore mortality, including wolf mortality, is caused by humans in Scandinavia^22,23,55^ and elsewhere^56,57^, it seems reasonable to argue that avoiding humans could be an advantageous trait and subject for selection either as an innate behavior, behavior learned by the parents, or a combination thereof.

Our results indicating that wolves born in natal territories with high degree of anthropogenic influence (Fig. 3) tended to avoid human-related features within their adult home ranges more than wolves born in more remote areas (Tables 1, 2; Figs 4, 5) suggest that learning during the natal stage may play a role for the behavioral decisions of wolves later in life. Such patterns have not been documented earlier, to our knowledge, yet it might be expected in an animal with cognitive abilities, such as the wolf. It has been shown that brown bears *Ursus arctos* display human avoidance at all spatial and temporal scales^24^, sometimes in clear response to specific human activities. For instance, bears become more nocturnal when annual hunting seasons start^58^ and right after encountering humans in the forest^59^. Similar experiences may also explain why individual wolves born in areas with higher degree of anthropogenic influence tended to avoid human features within their established home range more than wolves born in areas less influenced by humans, where they likely had less chances to interact with humans and thus to learn from their own experiences.

The results suggested that within wolf territories, the movement of wolf pairs relative to human structures showed a more consistent relationship with the characteristics of the natal habitat of the female than that of the male. That is, wolf pairs composed of a female born in an area characterized by a high degree of anthropogenic influence tended to avoid areas close to humans (Tables 1 and 2). Within wolf territories, the territorial pair mostly moves together, except during the denning period^60^. However, the specific role of the male and the female in habitat selection is not known. Among birds, nest site selection by great tit *Parus major* pairs is influenced by early experiences of the male, but not the female^7,61^. In a mammal species (the Siberian flying squirrel *Pteromys volan;* Selonen *et al*.^62^), female space use seemed to be related to the availability of food resources, breeding behavior and/or the defense of offspring, while male space use was largely determined by the spatiotemporal distribution of mates. In addition, females seemed to be more cautious than males. For instance, female orcas *Orcinus orcas* are particularly cautious when approaching humans^63^, and female Siberian flying squirrels occupy and move mostly in single, suitable patches of forest, whereas males, whose home ranges are larger, move around more fragmented terrain^64^. In Scandinavia, wolves, and especially females seemed to establish territories with habitat characteristics similar to those of their natal territories^19^. Wolf-pair habitat selection is likely a result of complex interactions between both pair members and their environment. Although the literature suggests gender specific behavior, our results do not allow us to draw any conclusions, but highlight the potential for interesting research on the role of each of the pair members in the habitat selection of the pair.

There are several possibilities to explain the statistical weakness of our results. We used spatial variables describing human features of the landscape as a proxy to characterize the degree of anthropogenic influence that wolves experienced in their natal territories. Other human-related factors experienced during the natal phase may also be important in shaping adult behavior. For example, exposure to stressful events in early life has effects on the adult behavior of an individual, as shown for both rodents e.g.^65^ and humans e.g.^66^. For wolves, some stressful events at an early life-stage, (e.g., direct encounters with people or sudden increases in human disturbance because of hunting), could be important in shaping adult wolf behavior. Therefore, our variables characterizing human exposure may have been too coarse to find a clear pattern, and/or there might be confounding factors that we could not incorporate at the right scale in our analyses. For example, wolves breed during the spring-summer, which is likely influencing their behavior more than any other factor at that time of the year, e.g., females stay at the breeding site most of the time while rearing pups^67^. Moose is the staple prey for Scandinavian wolves^37^. Thus, the local distribution of moose in a given time period might be a key driver of wolf habitat selection. In winter, moose temporally aggregate in parts of central Scandinavia, reaching densities as high as 5–6 moose/km^2^ in low-elevation wintering areas when snow cover gets very deep at higher altitudes^68^. If moose are close to plowed roads, railways, or settlements, for instance, wolves may select those areas to hunt efficiently. We used moose density at the wolf territory level as proxy of the potential influence of prey in wolf habitat selection, but we did not have access to a variable describing moose distribution at a finer spatial scale.

The occurrence of NHPI may help understand and predict habitat selection and habitat use at different habitat scales, and this is a timely need now that large carnivores are recolonizing former ranges^33^. Movements close to human infrastructure increases mortality risk for large carnivores^69^, potentially generating ecological traps where survival and therefore fitness are lower close to human settlements and roads than in remoter areas (e.g., Penteriani *et al*.^70^). Indeed, this might be a plausible reason why we found some support for our hypothesis 1 (Fig. 1). Our study was conducted on request of a management agency to better understand why some wolves seemed to be close to settlements in Scandinavia (Miljødirektoratet^27^). In this context, finding that wolves experiencing a higher degree of human encroachment early in life tended to avoid human features during adulthood suggests that settlements are not luring wolves to their proximity in Scandinavia, which is a comforting finding. Nevertheless, wolf research and management authorities must continue monitoring the situation given the statistically weak support of our results and the ongoing recovery of the wolf population in Scandinavia^26^, which may progressively expose some individual wolves to areas with higher human encroachment.

We suggest that our methodological approach is useful for wolves and other species in Scandinavia and elsewhere. The Scandinavian landscape is more homogeneous and has a lower human density than central and southern Europe, for instance^19^. Therefore, the apparent pattern that we have found in Scandinavian wolves may be different in scenarios with more contrasting levels of human encroachment. As previously noted, the lack of research on the influence of natal experience on habitat selection later in life, and at third and fourth order, is striking^13^. We hope our study will stimulate more research on the potential importance of early-life experience for large carnivore habitat selection, given the implications that this can have for conservation and management of such species and those they interact with, including us humans. Moreover, the global ongoing increase in human population size and human-caused habitat fragmentation is likely to continue, and wildlife is expected to face increased challenges. Better knowledge of NHPI may be crucial in management of human-animal interactions, and for the long-term survival and conservation of species, populations and habitat diversity in this increasingly human-dominated world.

## Supplementary information

Supplementary Information
