# Supplementary material for: Testing the influence of habitat experienced during the natal phase on habitat selection later in life in Scandinavian wolves
Source: Sci Rep. 2019 Apr 25;9:6526. doi: 10.1038/s41598-019-42835-1 (PMC6484024; doi:10.1038/s41598-019-42835-1)
Supplement: Supplementary file 1 — Supplementary Information [file 41598_2019_42835_MOESM1_ESM.docx]

**Supplementary Information**

**Testing the influence of habitat experienced during the natal phase on habitat selection later in life in Scandinavian wolves**

Cyril Milleret, Andrés Ordiz, Ana Sanz-Pérez, Antonio Uzal, David Carricondo-Sanchez, Ane Eriksen, Håkan Sand, Petter Wabakken, Camilla Wikenros, Mikael Åkesson, Barbara Zimmermann

**Supplementary Information 1**

Summary of the wolf GPS locations used.

**Table. S1.1** List of GPS data used for the analysis. Start and End denote the start and end date for which GPS locations were available. Range shows the number of days for which GPS locations were available. “W” and “S” denote territories monitored during the winter and summer, respectively.

| Territory_year | Start | End | Range | Locations | Locations moving |
| --- | --- | --- | --- | --- | --- |
| Jangen_2004_W | 2/2/2004 | 4/1/2004 | 60 | 1898 | 776 |
| Kloten_2008_W | 2/11/2008 | 3/31/2008 | 50 | 1120 | 427 |
| Kukumaki_2014_S | 5/19/2014 | 6/22/2014 | 35 | 721 | 273 |
| Nyskoga_2003_S | 6/2/2003 | 6/9/2003 | 8 | 184 | 143 |
| Nyskoga_2004_W | 2/13/2004 | 3/16/2004 | 33 | 1454 | 476 |
| Riala_2010_W | 2/22/2010 | 4/4/2010 | 42 | 1590 | 577 |
| Stadra_2003_W | 1/27/2003 | 3/17/2003 | 50 | 1645 | 369 |
| Tandsjon_2014_S | 5/19/2014 | 6/21/2014 | 33 | 695 | 220 |
| Tandsjon_2014_W | 3/19/2014 | 4/25/2014 | 37 | 650 | 243 |
| Tenskog_2010_W | 2/13/2010 | 4/11/2010 | 57 | 1432 | 503 |
| Ulriksberg_2006_W | 2/16/2006 | 4/13/2006 | 57 | 1233 | 456 |
| Kukumaki_2014_W | 3/3/2014 | 4/25/2014 | 54 | 934 | 373 |
| Bograngen_2003_S | 6/2/2003 | 7/13/2003 | 42 | 1594 | 608 |
| Fulufjallet_2009_W | 2/15/2009 | 4/8/2009 | 53 | 314 | 115 |
| Glaskogen_2002_S | 6/24/2002 | 7/31/2002 | 38 | 777 | 296 |
| Kukumaki_2015_W | 3/4/2015 | 4/24/2015 | 51 | 1823 | 1119 |
| Kukumaki_2015_S | 5/10/2015 | 6/30/2015 | 51 | 3514 | 3214 |
| Fulufjallet_2010_W | 4/1/2010 | 4/30/2010 | 30 | 1419 | 1034 |
| Fulufjallet_2010_S | 5/1/2010 | 6/1/2010 | 32 | 1517 | 1206 |
| Tandsjon_2012_W | 2/20/2012 | 4/30/2012 | 71 | 163 | 107 |
| Tandsjon_2012_S | 5/1/2012 | 5/14/2012 | 14 | 82 | 30 |
| Tenskog_2011_W | 3/14/2011 | 4/30/2011 | 48 | 1251 | 534 |
| Tenskog_2011_S | 5/1/2011 | 6/26/2011 | 57 | 1965 | 1012 |
| Kukumaki_2013_W | 2/25/2013 | 4/28/2013 | 63 | 1115 | 513 |
| Tyngsjo_2002_W | 1/31/2002 | 4/24/2002 | 84 | 1872 | 771 |
| Bograngen_2003_W | 2/17/2003 | 4/20/2003 | 63 | 2862 | 886 |
| Grafjell_2001_W | 12/11/2001 | 12/31/2001 | 21 | 927 | 462 |
| Grafjell_2002_W | 1/1/2002 | 4/21/2002 | 111 | 4080 | 2098 |
| Grafjell_2003_W | 2/17/2003 | 4/20/2003 | 63 | 2471 | 1179 |
| Grafjell_2003_S | 6/2/2003 | 7/14/2003 | 43 | 1571 | 1081 |
| Djurskog_2004_S | 6/21/2004 | 7/12/2004 | 22 | 773 | 306 |
| Koppang_2004_S | 6/14/2004 | 7/5/2004 | 22 | 1655 | 1199 |
| Ulriksberg_2007_W | 1/15/2007 | 3/9/2007 | 54 | 1227 | 487 |
| Grasmark_2007_W | 2/18/2007 | 4/9/2007 | 51 | 1333 | 606 |
| Kloten_2009_S | 6/13/2009 | 7/11/2009 | 29 | 2049 | 1447 |
| Hasselfors_2003_W | 2/9/2003 | 4/13/2003 | 64 | 2047 | 936 |
| Grafjell_2004_S | 6/14/2004 | 7/5/2004 | 22 | 408 | 89 |
| Halgan_2003_S | 6/21/2003 | 7/31/2003 | 40 | 705 | 332 |
| Djurskog_2004_W | 2/1/2004 | 3/28/2004 | 57 | 121 | 68 |
| Uttersberg_2005_W | 12/1/2005 | 12/31/2005 | 31 | 631 | 232 |
| Uttersberg_2006_W | 1/1/2006 | 12/17/2006 | 351 | 981 | 339 |
| Julussa_2015_S | 6/2/2015 | 7/23/2015 | 51 | 910 | 341 |
| Aspafallet_2015_W | 1/31/2015 | 4/30/2015 | 90 | 2152 | 804 |
| Aspafallet_2015_S | 5/1/2015 | 7/31/2015 | 91 | 2355 | 984 |

**Supplementary Information 2**

Summary of spatial covariates used to explain wolf habitat selection and degree of anthropogenic influence within natal territory.

**Table S2.1.** List of covariates used to model habitat selection of wolf pairs in Central Scandinavia from 2001-2015. The variable “Mire” was correlated with “Forest” and was not included in the model (r > 0.6). “Distance to main human features” and “Distance to all human features” were correlated with “buildings” (r > 0.6) and “secondary roads” (r > 0.6), respectively. Therefore, we did not use “buildings” and 2secondary roads” when “Distance to main human features” and “Distance to all human features” were used, respectively.

| **Name** | **Description** | **Source** |
| --- | --- | --- |
| Forest | Proportion of forest within 25 x 25 m cell | Swedish Corine land cover map Lantmäteriet, Sweden, merged with Northern Research Institute’s vegetation map, Norway |
| Elevation | Elevation (m.) | DEM 25 x 25 m; Geographical Data  Sweden, Lantmäteriet; Norge digital, Statens kartverk, Norway |
| Mire | Proportion of mires within 25 x 25 m cell | Swedish Corine land cover map Lantmäteriet, Sweden, 25 x 25 m merged with Northern Research Institute’s vegetation map, Norway, 30 x 30 m into a 25 x 25 m raster. |
| TRI | Terrain ruggedness index | DEM 25 x 25 m; Geographical Data  Sweden, Lantmäteriet; Norge digital, Statens kartverk, Norway^71^ |
| Distance Main roads | Distance from main roads (m.). | (1:100 000, Lantmäteriet,Sweden; N50 kartdata,Staten-skartverk, Norway) |
| Distance Secondary roads | Distance from secondary roads (m.). | (1:100 000, Lantmäteriet,Sweden; N50 kartdata,Staten-skartverk, Norway) |
| Distance Buildings | Distance from buildings (m.) | (1:100 000, Lantmäteriet,Sweden; N50 kartdata,Staten-skartverk, Norway) |
| Distance to main human features | Distance from the closest main roads and buildings (m.) | (1:100 000, Lantmäteriet,Sweden; N50 kartdata,Staten-skartverk, Norway) |
| Distance to all human features | Distance from the closest main and gravel roads, and buildings (m.) | (1:100 000, Lantmäteriet,Sweden; N50 kartdata,Staten-skartverk, Norway) |

**Table S2.2.** List of covariates used in a Principal Component Analysis to characterize the degree of anthropogenic influence to which wolves in Central Scandinavia (from 2001-2015) were exposed within their natal territory.

| **Name** | **Description** | **Source** |
| --- | --- | --- |
| Main roads | km of main roads per km^2^ | 1:100 000 Lantmäteriet, Sweden; N50 kartdata, Staten-skartverk, Norway |
| Second roads | km of gravel roads per km^2^ | 1:100 000 Lantmäteriet, Sweden; N50 kartdata, Staten-skartverk, Norway |
| Buildings | Number of buildings per km^2^ | 1:100 000 Lantmäteriet, Sweden; N50 kartdata, Staten-skartverk, Norway |
| Human density | Nº inhabitants per km^2^ | www.scb.se, Sweden; www.ssb.no, Norway |
| Agriculture | Proportion/absence of Agricultural areas | Swedish Corine land cover map Lantmäteriet, Sweden, 25 x 25 m merged with Northern Research Institute’s vegetation map, Norway, 30 x 30 m into a 25 x 25 m raster. |
| Anthropogenic areas | Proportion/absence of anthropogenic areas | Swedish Corine land cover map Lantmäteriet, Sweden, 25 x 25 m merged with Northern Research Institute’s vegetation map, Norway, 30 x 30 m into a 25 x 25 m raster. |
| Road buildings | Combination of building and road densities per km^2^ | ^23^ |

**Supplementary Information 3**

Results from the resource selection.

**Table S3.1**. Results from the resource selection functions performed on each individual wolf pair. Coefficients of selection are shown for the proportion of forest, elevation, Terrain Ruggedness index (TRI), distance to main roads (km^2^), distance to secondary roads (km^2^), and distance to main human features (km^2^) for all GPS locations. Minimum convex polygons were used to define availability. “W” and “S” denote territories monitored during the winter and summer, respectively.

| Study_year | (Intercept) | Forest | Elevation | TRI | Distance main roads | Distance second roads | Distance main human features |
| --- | --- | --- | --- | --- | --- | --- | --- |
| Jangen_2004_W | -2.90 | 0.07 | -0.31 | 0.20 | 0.54 | 0.16 | 0.38 |
| Kloten_2008_W | -2.94 | -0.15 | 0.38 | 0.10 | 0.15 | 0.25 | 0.32 |
| Kukumaki_2014_S | -3.07 | 0.62 | -0.67 | 0.32 | 0.05 | 0.57 | -0.01 |
| Nyskoga_2003_S | -3.51 | 0.68 | -0.90 | 0.38 | 1.27 | 0.30 | 0.11 |
| Nyskoga_2004_W | -3.12 | 0.57 | -0.87 | 0.19 | 0.52 | -0.01 | 0.02 |
| Riala_2010_W | -3.02 | 0.20 | 0.45 | 0.14 | 0.32 | 0.33 | 0.22 |
| Stadra_2003_W | -2.85 | 0.47 | 0.34 | -0.11 | -0.02 | 0.20 | -0.04 |
| Tandsjon_2014_S | -3.74 | 0.38 | 0.13 | 0.58 | -1.14 | -0.64 | 0.85 |
| Tandsjon_2014_W | -3.17 | 0.48 | -1.09 | -0.03 | 0.00 | -0.06 | 0.06 |
| Tenskog_2010_W | -3.06 | 0.17 | -0.53 | 0.72 | 0.58 | 0.38 | 0.05 |
| Ulriksberg_2006_W | -2.87 | 0.08 | -0.07 | 0.35 | -0.09 | -0.04 | 0.49 |
| Kukumaki_2014_W | -3.13 | 0.87 | -0.73 | 0.13 | -0.25 | -0.16 | 0.26 |
| Bograngen_2003_S | -2.79 | -0.08 | -0.05 | 0.23 | 0.11 | 0.06 | 0.33 |
| Fulufjallet_2009_W | -3.18 | 0.63 | -1.29 | 0.27 | 0.58 | -0.25 | 0.29 |
| Glaskogen_2002_S | -3.07 | 0.44 | 0.52 | -0.23 | 0.33 | -0.04 | 0.03 |
| Kukumaki_2015_W | -2.93 | 0.28 | -0.63 | 0.38 | 0.18 | -0.36 | 0.19 |
| Kukumaki_2015_S | -3.11 | 0.47 | 0.40 | 0.18 | 0.14 | 0.07 | 0.44 |
| Fulufjallet_2010_W | -2.95 | 0.54 | -0.80 | 0.19 | 0.07 | 0.08 | 0.35 |
| Fulufjallet_2010_S | -3.33 | 0.64 | 0.56 | 0.27 | -1.06 | -0.72 | 1.06 |
| Tandsjon_2012_W | -3.00 | 0.36 | -0.53 | 0.09 | -0.26 | -0.23 | 0.22 |
| Tandsjon_2012_S | -3.96 | 0.47 | 0.05 | -0.05 | -0.55 | -1.46 | 1.22 |
| Tenskog_2011_W | -3.02 | 0.11 | -0.54 | 0.77 | 0.15 | 0.06 | 0.16 |
| Tenskog_2011_S | -3.28 | 0.43 | 0.13 | 0.74 | 0.39 | -0.24 | -0.17 |
| Kukumaki_2013_W | -3.15 | 0.21 | -1.26 | 0.18 | 0.41 | -0.13 | 0.11 |
| Tyngsjo_2002_W | -2.96 | 0.31 | 0.05 | 0.15 | 0.43 | 0.23 | 0.26 |
| Bograngen_2003_W | -2.82 | 0.04 | -0.35 | 0.25 | -0.07 | 0.35 | 0.32 |
| Grafjell_2001_W | -2.93 | -0.01 | -0.61 | 0.50 | 0.01 | -0.15 | 0.35 |
| Grafjell_2002_W | -3.31 | -0.11 | -1.61 | 0.80 | 0.32 | 0.15 | 0.38 |
| Grafjell_2003_W | -3.63 | 0.02 | -1.96 | 0.34 | -0.01 | 0.02 | 0.52 |
| Grafjell_2003_S | -3.33 | -0.52 | 0.27 | 0.83 | 0.49 | 0.22 | -0.16 |
| Djurskog_2004_S | -3.06 | 0.23 | 0.52 | 0.32 | 0.43 | 0.19 | -0.54 |
| Koppang_2004_S | -3.93 | 0.34 | 0.22 | 0.53 | 1.41 | 0.71 | 0.13 |
| Ulriksberg_2007_W | -2.78 | 0.28 | -0.27 | 0.13 | -0.15 | -0.01 | 0.26 |
| Grasmark_2007_W | -2.85 | 0.12 | -0.06 | 0.01 | -0.14 | -0.53 | 0.34 |
| Kloten_2009_S | -3.01 | 0.30 | 0.45 | -0.64 | 0.16 | -0.03 | 0.22 |
| Hasselfors_2003_W | -3.00 | 0.53 | 0.16 | -0.01 | 0.50 | -0.05 | 0.11 |
| Grafjell_2004_S | -3.17 | 0.37 | 0.49 | -0.73 | -0.13 | -0.48 | 0.65 |
| Halgan_2003_S | -2.80 | 0.35 | 0.04 | -0.04 | 0.15 | 0.22 | 0.12 |
| Djurskog_2004_W | -3.19 | 0.04 | 0.47 | 0.18 | 0.70 | -0.20 | 0.12 |
| Uttersberg_2005_W | -2.89 | 0.42 | -0.45 | 0.03 | -0.21 | -0.12 | 0.58 |
| Uttersberg_2006_W | -2.84 | 0.39 | -0.27 | -0.04 | -0.13 | 0.14 | 0.44 |
| Julussa_2015_S | -3.22 | 0.64 | 0.13 | 0.59 | 0.00 | -0.11 | 0.49 |
| Aspafallet_2015_W | -2.93 | 0.25 | -0.04 | 0.08 | -0.30 | -0.02 | 0.76 |
| Aspafallet_2015_S | -3.01 | 0.38 | 0.02 | -0.24 | -0.46 | 0.17 | 0.76 |

**Supplementary Information 4**

Results from the linear mixed effect models on the RSF value obtained for each individual and their degree of anthropogenic influence within their natal territory.

**Table S4.1.** Coefficient estimates when considering GPS locations while traveling and distance to all humans features by defining availability using minimum convex polygon.

|  | **Beta** | **SE** | **CI Low** | **CI high** |
| --- | --- | --- | --- | --- |
|  |  | **Null** |  |  |
| (Intercept) | 0.13 | 0.14 | 0.05 | 0.25 |
| moose_M | -0.71 | 0.47 | -1.31 | 0.31 |
| **Natal_F** | | | | |
| (Intercept) | 0.14 | 0.14 | -0.13 | 0.42 |
| moose_M | -0.68 | 0.48 | -1.67 | 0.27 |
| PC_F | 0.03 | 0.05 | -0.06 | 0.12 |
|  |  | **Natal_F * Season** |  |  |
| (Intercept) | 0.15 | 0.14 | -0.11 | 0.41 |
| moose_M | -0.61 | 0.43 | -1.53 | 0.19 |
| PC_F | 0 | 0.06 | -0.11 | 0.1 |
| Season_FW | -0.03 | 0.1 | -0.25 | 0.14 |
| PC_F:Season_FW | 0.05 | 0.07 | -0.07 | 0.2 |
|  |  | **Natal_M** |  |  |
| (Intercept) | 0.15 | 0.15 | -0.16 | 0.47 |
| moose_M | -0.77 | 0.52 | -1.85 | 0.27 |
| PC_M | 0 | 0.03 | -0.06 | 0.05 |
| **Natal_M * Season** | | | | |
| (Intercept) | 0.18 | 0.15 | -0.11 | 0.47 |
| moose_M | -0.67 | 0.47 | -1.69 | 0.21 |
| PC_M | -0.02 | 0.03 | -0.08 | 0.04 |
| Season_MW | -0.09 | 0.08 | -0.29 | 0.06 |
| PC_M:Season_MW | 0.04 | 0.04 | -0.04 | 0.12 |
|  |  | **Natal_M+Natal_F** |  |  |
| (Intercept) | 0.18 | 0.16 | -0.13 | 0.48 |
| moose_M | -0.78 | 0.52 | -1.79 | 0.25 |
| PC_M | -0.01 | 0.03 | -0.07 | 0.05 |
| PC_F | 0.04 | 0.05 | -0.05 | 0.13 |
| **Natal_M*Natal_F** | | | | |
| (Intercept) | 0.2 | 0.16 | -0.12 | 0.51 |
| moose_M | -0.8 | 0.54 | -1.82 | 0.23 |
| PC_M | -0.01 | 0.03 | -0.07 | 0.05 |
| PC_F | 0.06 | 0.06 | -0.05 | 0.16 |
| PC_M:PC_F | -0.01 | 0.02 | -0.03 | 0.02 |
|  |  |  |  |  |

**Table S4.2**. Coefficient estimates when considering GPS locations while traveling and distance to main humans features by defining availability using kernel home range.

|  | **Beta** | **SE** | **CI Low** | **CI high** |
| --- | --- | --- | --- | --- |
|  |  | **Null** |  |  |
| (Intercept) | 0.21 | 0.08 | 0.05 | 0.3 |
| moose_M | -0.21 | 0.28 | -0.55 | 0.15 |
|  |  | **Natal_F** |  |  |
| (Intercept) | 0.23 | 0.08 | 0.07 | 0.39 |
| moose_M | -0.18 | 0.27 | -0.71 | 0.34 |
| PC_F | 0.04 | 0.03 | -0.01 | 0.1 |
| **Natal_F * Season** | | | | |
| (Intercept) | 0.25 | 0.1 | 0.06 | 0.44 |
| moose_M | -0.23 | 0.28 | -0.75 | 0.3 |
| PC_F | 0.02 | 0.04 | -0.07 | 0.1 |
| Season_FW | 0 | 0.09 | -0.16 | 0.16 |
| PC_F:Season_FW | 0.05 | 0.06 | -0.07 | 0.16 |
|  | | **Natal_M** |  |  |
| (Intercept) | 0.2 | 0.09 | 0.03 | 0.37 |
| moose_M | -0.19 | 0.3 | -0.75 | 0.37 |
| PC_M | 0 | 0.02 | -0.03 | 0.04 |
| **Natal_M * Season** | | | | |
| (Intercept) | 0.23 | 0.11 | 0.02 | 0.43 |
| moose_M | -0.21 | 0.31 | -0.78 | 0.36 |
| PC_M | 0.01 | 0.03 | -0.04 | 0.07 |
| Season_MW | -0.03 | 0.08 | -0.18 | 0.12 |
| PC_M:Season_MW | -0.02 | 0.04 | -0.09 | 0.06 |
| **Natal_M+Natal_F** | | | | |
| (Intercept) | 0.25 | 0.09 | 0.07 | 0.43 |
| moose_M | -0.22 | 0.29 | -0.77 | 0.33 |
| PC_M | -0.01 | 0.02 | -0.05 | 0.03 |
| PC_F | 0.05 | 0.03 | -0.01 | 0.12 |
|  | | **Natal_M*Natal_F** |  |  |
| (Intercept) | 0.25 | 0.1 | 0.07 | 0.44 |
| moose_M | -0.22 | 0.3 | -0.77 | 0.32 |
| PC_M | -0.01 | 0.02 | -0.05 | 0.03 |
| PC_F | 0.05 | 0.04 | -0.02 | 0.12 |
| PC_M:PC_F | 0 | 0.01 | -0.02 | 0.02 |
|  | |  |  |  |

**Table S4.3.** Coefficient estimates when considering all GPS locations and distance to all human features by defining availability using minimum convex polygon..

|  | **Beta** | **SE** | **CI Low** | **CI high** |
| --- | --- | --- | --- | --- |
|  |  | **Null** |  |  |
| (Intercept) | 0.26 | 0.15 | 0.10 | 0.41 |
| moose_M | -1.06 | 0.5 | -1.41 | 0.31 |
|  |  | **Natal_F** |  |  |
| (Intercept) | 0.26 | 0.15 | -0.04 | 0.57 |
| moose_M | -1.09 | 0.53 | -2.18 | -0.04 |
| PC_F | 0 | 0.05 | -0.09 | 0.09 |
|  |  | **Natal_F * Season** |  |  |
| (Intercept) | 0.29 | 0.15 | 0.01 | 0.59 |
| moose_M | -1.11 | 0.49 | -2.13 | -0.19 |
| PC_F | -0.07 | 0.06 | -0.18 | 0.03 |
| Season_FW | -0.03 | 0.09 | -0.23 | 0.14 |
| PC_F:Season_FW | 0.12 | 0.06 | 0.03 | 0.23 |
|  | | **Natal_M** |  |  |
| (Intercept) | 0.28 | 0.16 | -0.04 | 0.62 |
| moose_M | -1.14 | 0.55 | -2.27 | -0.04 |
| PC_M | -0.01 | 0.03 | -0.06 | 0.05 |
| **Natal_M * Season** | | | | |
| (Intercept) | 0.34 | 0.16 | 0.04 | 0.67 |
| moose_M | -1.09 | 0.51 | -2.15 | -0.12 |
| PC_M | -0.03 | 0.03 | -0.09 | 0.04 |
| Season_MW | -0.13 | 0.09 | -0.32 | 0.04 |
| PC_M:Season_MW | 0.04 | 0.04 | -0.03 | 0.12 |
|  | | **Natal_M+Natal_F** |  |  |
| (Intercept) | 0.29 | 0.17 | -0.05 | 0.62 |
| moose_M | -1.16 | 0.57 | -2.27 | -0.04 |
| PC_M | -0.01 | 0.03 | -0.07 | 0.05 |
| PC_F | 0 | 0.06 | -0.1 | 0.1 |
|  | | **Natal_M*Natal_F** |  |  |
| (Intercept) | 0.3 | 0.17 | -0.03 | 0.63 |
| moose_M | -1.16 | 0.58 | -2.25 | -0.06 |
| PC_M | 0 | 0.03 | -0.07 | 0.06 |
| PC_F | 0.02 | 0.06 | -0.09 | 0.13 |
| PC_M:PC_F | -0.01 | 0.02 | -0.04 | 0.02 |
|  | |  |  |  |

**Table S4.4**. Coefficient estimates when considering all GPS locations and distance to all human features by defining availability using kernel home range.

|  | **Beta** | **SE** | **CI Low** | **CI high** |
| --- | --- | --- | --- | --- |
|  |  | **Null** |  |  |
| (Intercept) | 0.28 | 0.13 | 0.05 | 0.45 |
| moose_M | -0.37 | 0.43 | -1.26 | 0.26 |
|  |  | **Natal_F** |  |  |
| (Intercept) | 0.28 | 0.13 | 0.04 | 0.51 |
| moose_M | -0.3 | 0.44 | -1.13 | 0.52 |
| PC_F | 0.03 | 0.04 | -0.05 | 0.11 |
|  |  | Natal_F * Season |  |  |
| (Intercept) | 0.26 | 0.14 | 0.01 | 0.51 |
| moose_M | -0.29 | 0.44 | -1.12 | 0.53 |
| PC_F | 0.03 | 0.05 | -0.06 | 0.13 |
| Season_FW | 0.03 | 0.09 | -0.12 | 0.2 |
| PC_F:Season_FW | 0 | 0.06 | -0.11 | 0.12 |
|  | | Natal_M |  |  |
| (Intercept) | 0.3 | 0.14 | 0.03 | 0.56 |
| moose_M | -0.42 | 0.47 | -1.3 | 0.46 |
| PC_M | -0.01 | 0.02 | -0.06 | 0.04 |
| **Natal_M * Season** | | | | |
| (Intercept) | 0.28 | 0.15 | 0 | 0.56 |
| moose_M | -0.4 | 0.47 | -1.28 | 0.46 |
| PC_M | 0 | 0.03 | -0.06 | 0.05 |
| Season_MW | 0.02 | 0.08 | -0.11 | 0.18 |
| PC_M:Season_MW | -0.01 | 0.04 | -0.08 | 0.06 |
|  | | **Natal_M+Natal_F** |  |  |
| (Intercept) | 0.31 | 0.14 | 0.06 | 0.57 |
| moose_M | -0.38 | 0.46 | -1.23 | 0.46 |
| PC_M | -0.02 | 0.03 | -0.07 | 0.03 |
| PC_F | 0.04 | 0.05 | -0.04 | 0.13 |
|  | | Natal_M*Natal_F |  |  |
| (Intercept) | 0.32 | 0.14 | 0.06 | 0.57 |
| moose_M | -0.38 | 0.47 | -1.23 | 0.46 |
| PC_M | -0.02 | 0.03 | -0.07 | 0.03 |
| PC_F | 0.04 | 0.05 | -0.05 | 0.13 |
| PC_M:PC_F | 0 | 0.01 | -0.02 | 0.02 |
|  | |  |  |  |
